# Severe Reversible Heart Failure Enhanced by Sorafenib Treatment for Hepatocellular Carcinoma

**DOI:** 10.7759/cureus.76993

**Published:** 2025-01-06

**Authors:** Luís Guilherme Santos, Ricardo Roque, Rita Antunes Santos, Catarina Neves, Nuno Bonito

**Affiliations:** 1 Medical Oncology, Instituto Português de Oncologia de Coimbra Francisco Gentil, Coimbra, PRT; 2 Cardiology, Instituto Português de Oncologia de Coimbra Francisco Gentil, Coimbra, PRT

**Keywords:** atrial fibrillation (af), cardiotoxicity, heart failure, hepatocellular carcinoma, sorafenib

## Abstract

The multitarget oral tyrosine kinase inhibitor sorafenib is an effective first-line treatment option in unresectable hepatocellular carcinoma. Through its mechanism of action, it has been associated with cardiotoxicity, mainly hypertension, which is usually low-grade and well-managed with behavioral changes and antihypertensor treatment adjustment, if needed. Acute, symptomatic heart failure is rarely described. We present the case of a patient with priors of arterial hypertension, dyslipidemia, type 2 diabetes mellitus, and non-alcoholic steatohepatitis-related cirrhosis of the liver, with the diagnosis of hepatocellular carcinoma treated with sorafenib, with previous excellent tolerability and stable disease. Dyspnea and detection of atrial fibrillation with severe reduction of left ventricular ejection fraction (26%), three years after the beginning of treatment, led to the diagnosis of acute heart failure with reduced ejection fraction and class IV New York Heart Association symptoms, confirmed to be enhanced by sorafenib, and partially reversible after its suspension and optimization of cardiological treatment. A multidisciplinary approach, prompt recognition, and aggressive treatment of this rare and severe toxicity are essential in determining a favorable outcome.

## Introduction

Sorafenib, a multitarget oral tyrosine kinase inhibitor (TKI), is a staple in the first-line treatment of advanced hepatocellular carcinoma since its proven benefit in overall survival on the SHARP (10.7 versus 7.9 months) and ORIENTAL (6.5 versus 4.2 months) phase III randomized controlled trials [1,2] established it as a standard of care. Sorafenib inhibits multiple pathways involved in tumor growth and angiogenesis, such as vascular endothelial growth factor receptor 2/3 (VEGFR 2/3) and platelet-derived growth factor receptor (PDGFR), and has been linked to cardiotoxicity, mainly arterial hypertension and atrial fibrillation (AF). Severe symptomatic heart failure is a serious adverse event, only rarely described (incidence as low as 4%) [3]. When present, it tends to appear within the first 30 days of sorafenib treatment and is usually associated with pre-existing cardiological conditions, which are often not properly compensated. While sorafenib-associated heart failure as late-onset toxicity is unusual, prompt detection and treatment of this adverse event are needed, as it adds to the challenging management of these patients and justifies the need for adequate long-term cardiac monitoring.

## Case presentation

**Figure 1:**
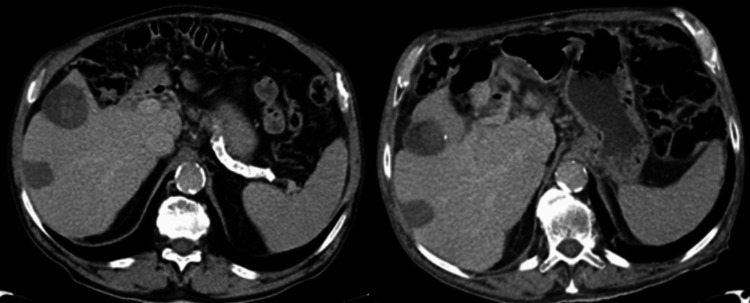
Abdominal computer tomography Abdominal computer tomography (CT) scan showing radiologically stable liver disease (no new lesions and no change in size of pre-existing lesions) at the beginning of sorafenib treatment (left) and three years later at the time of symptomatic onset and sorafenib suspension (right).

We present the case of a 77-year-old male patient, Eastern Cooperative Oncology Group - Performance Scale (ECOG-PS) 0, with priors of non-alcoholic steatohepatitis (NASH)-related cirrhosis of the liver, arterial hypertension, dyslipidemia, and type 2 diabetes mellitus, previously treated with candesartan, hydrochlorothiazide, bisoprolol, atorvastatin, metformin, and sitagliptin. The patient was in active first-line treatment with sorafenib (400 mg twice daily) for steatohepatitic variant hepatocellular carcinoma, diagnosed approximately three years before and subject to previous thermoablation. The patient had radiological stable disease at each response assessment since the beginning of treatment (Figure 1). The treatment has been well tolerated with clear clinical benefits since its introduction. Previous cardiological studies through electrocardiogram (ECG) were always normal, with evidence of sinus rhythm, and no prior echocardiograms or other additional tests were performed.

**Figure 2:**
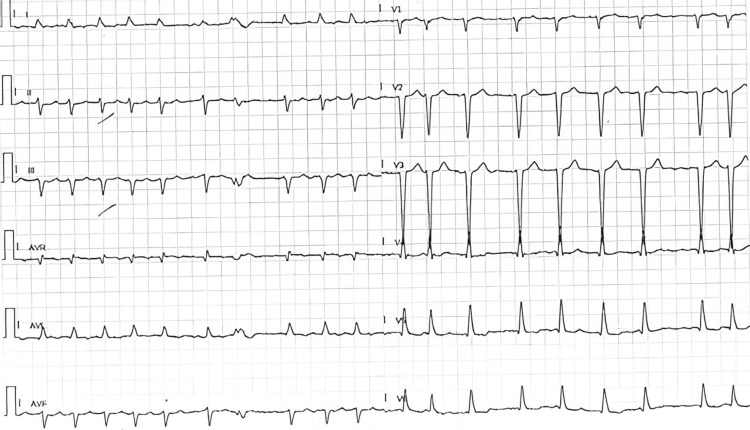
Electrocardiogram Electrocardiogram at time of symptomatic onset showing atrial fibrillation with rapid ventricular response.

The patient was admitted to the emergency department with symptoms of productive cough, dyspnea, orthopnea, and fever, with a subsequent diagnosis of previously undocumented pleural effusion concomitant with pneumonia and AF with rapid ventricular response (RVR) (Figure 2). The patient was discharged with antibiotics and apixaban. Of note, bisoprolol dosage was not adjusted, and conversion to sinus rhythm was not attempted at this moment despite evidence of de novo AF at the discretion of the emergency department team.

Two months later, the patient was again admitted with progressively worsening dyspnea and extreme fatigue, even with minimal exertion. At admission, physical examination showed right decreased ventricular murmur at pulmonary auscultation and bilateral edema of the lower limbs, with no other alterations. Blood gas was normal, and blood work showed a marked elevation of N-terminal pro-b-type natriuretic peptide (NT-proBNP) without other significant alterations. Chest radiography showed aggravated pleural effusion, this time requiring therapeutic thoracentesis, with symptomatic improvement. Cytological analysis of pleural liquid was compatible with a transudate showing no signs of neoplastic cells, excluding paraneoplastic cause as a possible sign of disease progression.

A second ECG confirmed AF with RVR. An echocardiogram was performed, revealing global left ventricular hypokinesia (mainly at the interventricular septum and inferior wall), with a severe decrease in left ventricular ejection fraction (LVEF), 26%, along with moderate aortic stenosis with low flow and low gradient. Patient was then referred to his cardio-oncology consult, and further tests were ordered: moderate aortic stenosis was confirmed by stress echocardiogram (which revealed absence of left ventricular contractile reserve, with stroke volume increase less than 20% and indexed aortic valve area of 0.76 cm^2^/m^2^) and aortic valve calcium-score CT scan by the Agatston method of 1,453, less than the limit for high probability of severe aortic stenosis (<1,600). Coronary angiography was performed to rule out coronary disease and was normal.

Despite heart rate control and intravenous diuretic therapy, NT-proBNP continued to rise, reaching a peak a week later. A diagnosis of acute heart failure with reduced ejection fraction and class IV New York Heart Association (NYHA) symptoms was then made.

Differential diagnosis focused on the cause of heart failure.

The patient had a recent diagnosis of pneumonia requiring antibiotics at symptomatic onset, but the initial clinical presentation was deemed unlikely to be compatible with septic shock by the ER team, and at second admission, infection-related signs and symptoms had subsided after antibiotherapy. This, however, could have been a cause for the onset of AF. ECG confirmed AF as a possible important cause of decompensation, which was not fully addressed at first decompensation. The echocardiogram showed signs of aortic stenosis, pointing to possible valvular heart failure, but severe aortic stenosis was ruled out by stress echocardiogram and calcium score evaluation. Hypothyroidism was ruled out (thyroid function tests were normal). Finally, and despite prolonged treatment with excellent previous tolerability and clinical benefit, sorafenib-induced cardiac toxicity (both AF and heart failure) could not be ruled out based on previously described clinical findings, NT-proBNP, and LVEF evolution profile.

The patient was started on intravenous furosemide, digoxin, dose-adjusted bisoprolol for rate control, and dapagliflozin, based on its proven benefits in reducing cardiovascular death in patients with heart failure. Apixaban was maintained. The re-introduction of candesartan and the introduction of spironolactone and sacubitril-valsartan were not initially successful as the patient maintained clinically significant hypotension, and it was not deemed safe. After a multidisciplinary meeting with the presence of a cardio-oncology specialist, it was considered that sorafenib could be perpetuating clinical heart failure, particularly due to the decrease in left ventricular contractility that continued despite the heart rate already being under control and the low contractile reserve seen on the stress echocardiogram. Heart failure was considered a Common Terminology Criteria for Adverse Events (CTCAE) grade 3, and sorafenib was suspended.

After treatment optimization and suspension of sorafenib, the patient remained hemodynamically stable with controlled blood pressure and heart rate without need for intravenous inotropic support and conversion to sinus rhythm, which was not attempted in the phase of greatest decompensation because the patient had severe atrial dilatation, which would lead to a low probability of success and because it would require a transoesophageal echocardiogram to rule out thrombi in the left atrial appendage, which was not easily available at that moment.

There was very significant clinical improvement (NYHA class improvement from IV to II), and the patient was only slightly symptomatic and with no clinical signs of fluid overload at the time of discharge. Re-evaluation echocardiogram showed signs of improved LVEF (31%) three weeks later. Blood pressure increased, allowing for the introduction of sacubitril/valsartan. Blood work revealed a marked decrease in NT-proBNP (Table 1 shows NT-proBNP value evolution through time). This evolutionary profile after sorafenib suspension suggests that sorafenib could have been indeed causing cardiotoxicity by aggravating the effect of tachycardia-induced cardiomyopathy in the context of AF.

**Table 1:** NT-proBNP value Evolution of NT-proBNP value through time NT-proBNP, N-terminal pro-b-type natriuretic peptide

Time of NT-proBNP determination and symptomatic correlation	Value	Reference value
NT-proBNP at onset of symptoms	5,630 pg/mL	<450 pg/mL for adults over 75 years of age
Peak NT-proBNP (two months after onset of symptoms - patient maintained NYHA class IV symptoms)	10,120 pg/mL	<450 pg/mL for adults over 75 years of age
NT-proBNP at first follow-up after sorafenib suspension (improvement from NYHA class IV to II and good quality of life)	1,299 ng/mL	<450 pg/mL for adults over 75 years of age

Sorafenib was permanently discontinued. Due to the impossibility of maintaining active oncological treatment coupled with the absence of evidence for disease progression and symptomatic improvement with the possibility of maintaining adequate quality of life, the patient and his family agreed to a palliative care referral. Currently, the patient still maintains a good performance status on NYHA class II symptoms. The latest echocardiogram showed continued improvement of LVEF (54%).

## Discussion

Sorafenib is a multi-target TKI, acting through suppression of multiple pathways such as rapid accelerated fibrosarcoma (RAF)/mitogen-activated protein kinase (MEK)/extracellular signal-regulated kinases (ERK) and inhibition of inhibition of VEGFR 2/3 and PDGFR. It has remained a valid first-line treatment option in unresectable hepatocellular carcinoma since the results of the SHARP and ORIENTAL trials demonstrated statistically significant overall survival benefit [1,2]. 

As is the case with other multikinase inhibitors with antiangiogenic effects, cardiotoxicity is a recognized adverse event of sorafenib, mainly consisting of hypertension, which is usually low-grade and well-managed when present. Heart failure as part of sorafenib cardiotoxicity is generally considered a rare event. In a study of patients treated with sorafenib for renal cell carcinoma, heart failure, defined as de novo elevation of NT-proBNP or decline in LVEF (both defining characteristics present in our case), was found in only 4% [3].

In a recent, large, retrospective pharmacovigilance study [4], which analyzed lenvatinib, sorafenib, cabozantinib, and regorafenib, heart failure was described as a class effect in all multikinase TKI’s but was found to be relatively less frequent with sorafenib compared to others such as lenvatinib and cabozantinib, while it might be more common when compared to regorafenib. Interestingly, however, according to the same publication, long-term use of sorafenib might increase the risk of heart failure when compared with lenvatinib and cabozantinib. Real incidence rates of heart failure as both short- and long-term toxicity, however, need to be confirmed by clinical trials. On the other hand and according to the same study [4], AF as an adverse event was more frequently detected with sorafenib, compared to lenvatinib or cabozantinib. Thus, in our case, AF as sorafenib-induced cardiotoxicity could also not be ruled out.

The mechanisms for TKI-induced heart failure are complex and not entirely understood [5]. Endothelial cell dysfunction through inhibition of VEGFR and RAS pathways, mitochondrial dysfunction, endoplasmic reticulum stress, dysregulated autophagy, and ferroptosis may all play a part in disrupting normal cardiovascular homeostasis, leading to toxicity. Raf-1 inhibition seems to be of particular importance with sorafenib, decreasing normal cardiomyocyte contractility and promoting apoptosis. Of note, hypertension (which is common with antiangiogenic TKIs) may lead to afterload stress on the heart, and normal adaptative mechanisms may be hindered by PDGFR inhibition [4]. Additionally [4], VEGFR inhibition may alter mechanisms regarding interaction between endothelial cells and cardiomyocytes. Both these factors can further contribute to the genesis of heart failure.

Significant, symptomatic heart failure in sorafenib-treated hepatocellular carcinoma continues to be rarely reported. Even though heart failure risk may increase with long-term sorafenib use [4], in previously reported cases [3,6,7], sorafenib-induced heart failure was always diagnosed shortly after treatment initiation (usually within the first 30 days of TKI use), highly contrasting to our case in which the diagnosis was made three years after sorafenib was introduced, with prior excellent tolerability. In these cases, and ours, patients often had priors of cardiac disease to some extent and improved after adequate compensation treatment and sorafenib suspension, highlighting that pre-existing cardiac disease and risk factors may play a part in increasing susceptibility to TKI-related cardiotoxicity. Data regarding the mechanism and real-life incidence of late-onset cardiac toxicity and the safety of treatment reintroduction after major events like these are lacking, highlighting the need for further studies.

## Conclusions

Sorafenib remains a valid option in first-line treatment for advanced hepatocellular carcinoma. Cases such as the one presented describe a recognized but rarely described and potentially very severe long-term adverse event of sorafenib treatment. Given the cardiotoxic potential of this drug, which may enhance or be enhanced by pre-existing cardiac conditions, this case highlights the importance of improving long-term cardiac vigilance strategies, optimizing management of previously known cardiac diseases (if present), and the importance of immediate and aggressive treatment, given the possibility of symptomatic reversion in these patients. A multidisciplinary approach, including oncology and cardio-oncology specialists, is of paramount importance due to the challenging complexity of these cases. Re-introduction of treatment in patients with such severe toxicities remains a topic of debate, and further studies on the matter are crucially needed.
